# Topics in machine learning for biomedical literature analysis and text retrieval

**DOI:** 10.1186/2041-1480-3-S3-S1

**Published:** 2012-10-05

**Authors:** Rezarta Islamaj Doğan, Lana Yeganova

**Affiliations:** 1 National Center for Biotechnology Information, National Library of Medicine, National Institutes of Health, Bethesda, Maryland, USA

## Introduction

Biomedical literature articles housed at the National Library of Medicine contain a wealth of scholarly knowledge of significant importance to researchers and health care professionals alike. This wealth of information is essential for researchers in order to build new hypothesis and to validate scientific discoveries and is essential for health care professionals in order to keep up-to-date with health related issues [1].

The ever-expanding volume of biomedical literature publications and other biomedical communications necessitates the work and study on developing better methods of efficiently accessing and retrieving relevant information from these textual resources. The digitizing of medical information particularly necessitates development of methods for efficient automatic text processing of medical and biomedical information. Automatic text processing has in its foundation natural language processing techniques, which combine linguistic knowledge and computer science theory to address the computational aspects of the task. Machine learning algorithms are heavily employed in these applications as is also experienced regularly in many other annual conference meetings.

The special session on "Machine Learning in Biomedical Literature Analysis and Text Retrieval" was held for the second time as part of the 10^th ^International Conference on Machine Learning and Applications, in Honolulu, Hawaii on December 18-21, 2011. The goal of this session was to present advancements in machine learning techniques that can improve the analysis of biomedical text.

In this supplement we present a collection of papers originally presented and published in the proceedings of the International Conference on Machine Learning and Applications (ICMLA 2011). These papers constitute an advance beyond the work originally presented at the conference and have gone through a separate rigorous review process.

Papers presented in this issue represent a wide cross-section of the type of work that goes on in machine learning today, with its focus on biomedical literature and clinical text. Kate [2] presents an unsupervised method which automatically induces a grammar and a parser for the sublanguage of a given genre of clinical reports from a corpus with no annotations. Author observes that clinical reports are written using a subset of natural language, and different genres of clinical reports use different sublanguages, which makes supervised training of a parser for clinical sentences very difficult. Ravikumar et al. [3] propose a method for automatic extraction of protein-specific residue mentions from the biomedical literature. They identify the amino acid residue mentions in the text using linguistic patterns and apply an automated graph-based method to learn syntactic patterns corresponding to protein-residue pairs. They demonstrate the effectiveness of distant supervision for automatic creation of training data for protein-residue relation extraction. Kim et al. [4] develop an unsupervised document clustering algorithm with a property that clusters are sufficiently explanatory for human understanding. For every cluster they extract subject terms and use them to describe the clusters. Yeganova et al. [5] study methods for automatically learning meaningful biomedical categories in Medline in an unsupervised fashion. They present methods for automatically extracting categories that are discussed in Medline. Rather than imposing external ontologies on Medline, they look for categories that emerge from the text. And, finally, Clematide et al. [6] present a method for extracting and raking the relations among different types of biomedical entities to make the curation process more efficient. Authors make use of existing resources such as Pharmogenomics Knowledge Base (PharmGKB) and the Comparative Toxicogenomics Database (CTD) to create a gold standard.

While covering a wide variety of topics, all papers in the supplement share one common characteristic - a shift from supervised methods towards semi-supervised and unsupervised methods. Authors agree that creating labeled training sets is extremely expensive and time-consuming, as they propose new and creative ways of automatically building training sets and demonstrate resourcefulness by using information from existing knowledge sources for compiling training data.

In conclusion, we thank the reviewers for their hard work and dedication to maintaining a professional review process. We also thank all authors of submitted papers for their diligences in responding to reviewers' comments.

## Competing interests

The authors declare that they have no competing interests.

## Authors' contributions

RID and LY are the special session co-chairs at ICMLA 2011 and contributed equally to the overall organization, reviewing and editing of this supplement on "Machine Learning for Biomedical Literature Analysis and Text Retrieval".
